# Low frequency of *CHEK2* 1100delC allele in Australian multiple-case breast cancer families: functional analysis in heterozygous individuals

**DOI:** 10.1038/sj.bjc.6602381

**Published:** 2005-02-08

**Authors:** C R Jekimovs, X Chen, J Arnold, M Gatei, D J Richard, kConFab Investigators, A B Spurdle, K K Khanna, G Chenevix-Trench

**Affiliations:** 1 Division of Cancer and Cell Biology, Queensland Institute of Medical Research, Post Office Royal Brisbane Hospital, Brisbane, QLD 4029, Australia; 2 Central Clinical Division, School of Medicine, University of Queensland, Brisbane, QLD 4072, Australia; 3 Peter MacCallum Cancer Centre, St Andrews Place, East Melbourne, VIC 3002, Australia

**Keywords:** familial breast cancer, *CHEK2*, germline variation, susceptibility, CHK2, DNA damage response

## Abstract

A protein-truncating variant of *CHEK2*, 1100delC, is associated with a moderate increase in breast cancer risk. We have determined the prevalence of this allele in index cases from 300 Australian multiple-case breast cancer families, 95% of which had been found to be negative for mutations in *BRCA1* and *BRCA2*. Only two (0.6%) index cases heterozygous for the *CHEK2* mutation were identified. All available relatives in these two families were genotyped, but there was no evidence of co-segregation between the *CHEK2* variant and breast cancer. Lymphoblastoid cell lines established from a heterozygous carrier contained approximately 20% of the *CHEK2* 1100delC mRNA relative to wild-type *CHEK2* transcript. However, no truncated CHK2 protein was detectable. Analyses of expression and phosphorylation of wild-type CHK2 suggest that the variant is likely to act by haploinsufficiency. Analysis of CDC25A degradation, a downstream target of CHK2, suggests that some compensation occurs to allow normal degradation of CDC25A. Such compensation of the 1100delC defect in *CHEK2* might explain the rather low breast cancer risk associated with the *CHEK2* variant, compared to that associated with truncating mutations in *BRCA1* or *BRCA2.*

The *CHEK2* tumour suppressor gene on chromosome 22q12.1 encodes a nuclear protein that is a member of the CDS1 subfamily of serine/threonine protein kinases. In response to ionising radiation (IR), threonine 68 of CHK2 is rapidly phosphorylated by ataxia telangiectasia mutated (ATM) (Matsuoka *et al*, 2000; Zhou *et al*, 2000), allowing oligomerisation and transautophosphorylation of CHK2 (Ahn *et al*, 2002; Xu *et al*, 2002). Activated CHK2 is involved in maintaining the G1/S and G2/M checkpoints by phosphorylation of CDC25A, CDC25C and p53 (Chaturvedi *et al*, 1999; Shieh *et al*, 2000; Falck *et al*, 2001), and repair of double-strand DNA breaks via homologous recombination (HR) through phosphorylation of BRCA1 (Lee *et al*, 2000). CHK2 is also involved in the induction of p53-dependent apoptosis through phosphorylation of p53 on Ser20 (Shieh *et al*, 2000), and, in a p53-independent manner, via phosphorylation of PML and E2F1 (Lin *et al*, 2001; Yang *et al*, 2002).

Somatic mutations of *CHEK2* are rare in breast tumours (Ingvarsson *et al*, 2002). However, a germline protein-truncating variant of *CHEK2*, 1100delC, was first identified in a patient with breast and colorectal cancer from a family with Li Fraumeni-like syndrome (Bell *et al*, 1999; Miller *et al*, 2002). This variant was subsequently found to be significantly associated with non-*BRCA1/2* familial breast cancer, being present in 5.1% of cases and only 1.1% of controls (*P*=0.00000003) (Meijers-Heijboer *et al*, 2002). Among families with male breast cancer, *CHEK2* 1100delC was present in 13.5% of cases (*P*=00015). Meijers-Heijboer *et al* (2002) estimated that the CHEK2 1100delC variant was associated with a two-fold increased risk of female breast cancer, and a 10-fold increased risk in men. However, additional studies of cohorts collected in Finland, USA and UK suggest that *CHEK2* 1100delC is not a risk factor for male breast cancer (Neuhausen *et al*, 2004; Syrjakoski *et al*, 2004). Nevertheless, the high frequency of *CHEK2* 1100delC in families with multiple cases of breast cancer and increased risk for female breast cancer has been substantiated by several additional studies (Vahteristo *et al*, 2002; Offit *et al*, 2003; Oldenburg *et al*, 2003). A consortium of nine case–control studies that included 9154 cases and 9881 controls has found that the *CHEK2* 1100delC variant is associated with a two-fold increased risk of female breast cancer (*P*=0.0000001) (*CHEK2* Breast Cancer Case-Control Consortium, 2004). The risk appears to be the highest for contralateral breast cancer in *CHEK2* 1100delC carriers who received radiation treatment for their first breast cancer (Broeks *et al*, 2004). In the Netherlands, *CHEK2* 1100delC was identified in 18% families with histories of both breast and colorectal in contrast to only 4% of breast cancer families without colorectal cancer. However, the suggestion that this mutant allele plays a role in susceptibility to both breast and colorectal cancer (Meijers-Heijboer *et al*, 2003) has not been substantiated in a study of patients with multiple colonic adenoma (Lipton *et al*, 2003).

The *CHEK2* 1100delC polymorphism results in a frameshift and premature protein truncation, resulting in the deletion of the kinase domain. The functional consequences of this deletion in a heterozygous cell line are unknown at present. In one previous study, a dramatically reduced expression of wild-type CHK2 was detected in an lymphoblastoid cell line (LCL) derived from an 1100delC carrier (Dong *et al*, 2003). Two other studies found markedly reduced expression of CHK2 in breast tumours from CHK2 carriers, although this was not always accompanied by loss of heterozygosity (LOH) at *CHEK2* (Vahteristo *et al*, 2002; Oldenburg *et al*, 2003). A significant proportion of sporadic breast cancers also demonstrate a similar loss of expression of CHK2, without carrying the *CHEK2* 1100delC variant (Sullivan *et al*, 2002). Here, we have assessed the frequency of *CHEK2* 1100delC variant among familial breast cancer cases from Australia and have analysed the expression, phosphorylation and activity of CHK2 in LCLs from heterozygous individuals.

## MATERIALS AND METHODS

### Multiple-case breast cancer families

Multiple-case breast cancer families were ascertained through The Kathleen Cuningham Consortium for Research into Familial Breast Cancer (kConFab: http://www.kconfab.org). The eligibility criteria for entry into kConFab for breast cancer families without a known pathogenic or splice site mutation in *BRCA1*, *BRCA2*, *PTEN* or *TP53* are as follows: Criterion 1 – four or more cases of breast or ovarian cancer, or Criterion 1B – two or three cases of breast or ovarian cancer if at least one of these cases is ‘high risk’ (male breast cancer, bilateral breast cancer, breast plus ovarian cancer, or breast cancer at less than 40 years of age). Both criteria require that two or more affected women are alive and that the families have four or more living, adult, female, unaffected first- or second-degree relatives of affected women; Criterion 4 – high-risk breast cancer families (as defined by the National Breast Cancer Centre Guidelines (http://www.nbcc.com.au)) from which fresh tissue is available but who do not fit other kConFab criteria. This study has the ethical committee approval from The Peter MacCallum Cancer Centre and the Queensland Institute of Medical Research.

The index case, defined as the youngest available affected individual in a family with blood available, was genotyped from a total of 283 multiple-case families (212 Criteria 1, 61 Criteria 1B and 10 Criterion 4), as well as 17 families that fitted Criteria 1 (*n*=9) or 1B (*n*=8) at the time of recruitment but who failed to fulfil the criteria when collection was complete because of deaths that occurred in the interim. In all, 286 out of 300 (95.3%) of these families had undergone testing for *BRCA1* and *BRCA2* mutations either by complete DNA sequencing (*n*=108), or by a variety of mutation detection methods (performed by the diagnostic laboratories) that were considered to be at least 80% sensitive (*n*=176). *BRCA1* and *BRCA2* testing is pending in the remaining 16 families. Seven families contained one or more cases of male breast cancer, 132 had one or more cases of bilateral female breast cancer and 152 had at least one case of colorectal cancer. Verification of all cancer diagnoses through medical records has been achieved for 51% of the reported tumours. Two archival paraffin blocks were available from *CHEK2* 1100delC carriers for LOH analysis.

### Genotyping

Genotyping was initially performed with the ABI PRISM 7700 (TaqMan) sequence detection system (Applied Biosystems, UK). A two-stage PCR procedure was used to avoid amplification of pseudogene sequences. Primers for the initial 537 bp PCR were (sequence differences between CHK2 and the pseudogene are shown in lowercase): forward: gCAAAaTTAAATGTCcTAACTTGC; reverse: GGCATGGTGGTGTGCatc. PCRs were carried out at 2 mM MgCl_2_ and 58°C annealing temperature, with 20 cycles. In all, 3 *μ*g of this PCR product was then used as template for the Taqman assay (Applied Biosystems, UK) using 450 nM primers (forward primer: AGTAGGTGGGGGTTCCACATAAG; reverse primer: GGCAGACTATGTTAATCTTTTTATTTTATGG) and an annealing temperature of 62°C. TAMRA probes, designed on the antisense strand, were used at 225 nM (C-allele (VIC): TGGAGTGCCCAAAATCAgTAATCTAAAATT) and 75 nM (delC-allele (FAM): TGGAGTGCCCAAAATCATAATCTAAAATTC) concentration.

### Loss of heterozygosity

Loss of heterozygosity was evaluated by macrodissection of the tumour using a haematoxylin and eosin-stained slide as guide, followed by direct sequencing using the outer primers. Loss of heterozygosity was scored by absence of the allele in the sequencing trace of the tumour, compared to its matching germline.

### Recombinant plasmids

Full-length CHK2 cDNA was cloned into pFLAG using the *Eco*R1 and *Hind*III sites. The 1100delC mutation was constructed using the Quick Change mutagenesis kit (Stratagene, USA) using pFLAG-CHK2 as a template with the following primers:

FOR_1100: 5′-GACTGTCTTATAAAGATTATGATTTTGGGCACTCCAAG-3′
REV_1100: 5′-CTTGGAGTGCCCAAAATCATAATCTTTATAAGACAGTC-3′

### Cell culture and transfections

The wild-type control LCL (98.004.0039), *CHEK2* 1100delC heterozygous (00.003.2070, 00.005.0442, 00.003.2053) LCLs and 293T fibroblasts were grown in RPMI 1640 with 10% foetal calf serum, 100 U ml^−1^ penicillin and 100 *μ*g ml^−1^ streptomycin at 37°C, 5% CO_2_.

293T fibroblasts were transfected with pFLAG or pFLAG-CHK2-1100delC using PEI, as described previously (Boussif *et al*, 1995), at 60–70% confluency and harvested 36 h post-transfection.

### RT–PCR and single-nucleotide primer extension analysis of 1100delC mRNA transcript

Total RNA was extracted from the control and mutant LCLs using the RNeasy Midi-kit (Qiagen, Australia). cDNA was transcribed using Superscript III reverse transcriptase (Promega, Australia) from 1 *μ*g total RNA primed with random hexamers. A 190 bp PCR product was amplified from the cDNA using the following primers: forward: CCAGATGCTCTTGGCTGTGC; reverse: TAGGTGGGGGTTCCACATAAGGT. The PCR product was sequenced directly using the forward primer. Single-nucleotide primer extension (SnuPE) reactions were carried out in a 50 *μ*l volume on 10 ng of purified PCR product using 10 pmol of either forward (GGACTGTCTTATAAAGATTA) or reverse (TCTTGGAGTGCCCAAAATCA) primers using 1.0 U of ampliTaq gold (Applied Biosystems, Australia) in 1 × PCR buffer and 1.6 mM MgCl_2_, and 1 *μ*l of ^32^P-dCTP, ^32^P-dTTP or ^32^P-dGTP (Amersham, Australia). For each PCR product, four different reactions were carried out in duplicate: two reactions with the forward primer with either ^32^P-dCTP or ^32^P-dTTP as the radiolabel, and two reactions for the reverse primer with either ^32^P-dGTP or ^32^P-dTTP as the radiolabel. The reactions were incubated at 95°C for 10 min, 55°C for 1 min, and 72°C for 2 min, after which 50 *μ*l of formamide-loading buffer was added to each reaction. The samples were then heated to 95°C for a further 5 min and 5 *μ*l loaded on a 6% sequencing gel, which was run at 1000 V for about 2 h, dried and exposed to film.

### Proteasome inhibition

The wild-type control (98.004.0039) and 1100delC heterozygous (00.003.2070) LCLs were treated with 10 *μ*g ml^−1^ *N*-acetyl-leucyl-leucyl-norleucinal (ALLN) (Sigma-Aldrich, Australia) in RPMI 1640 with 10% foetal calf serum, 100 U ml^−1^ penicillin and 100 *μ*g ml^−1^ streptomycin and grown at 37°C, 5% CO_2_. The cells were harvested at the indicated timepoints and protein extracts prepared.

### Protein extracts and Western blotting

Cells were mock or treated with 6 Gy of IR (^137^Cs) and harvested after 30 min. Cell extracts were prepared by lysis in Universal immunoprecipitation buffer (50 mM Tris–HCl, pH 7.4, 150 mM NaCl, 2 mM EDTA, 25 mM NaF, 25 mM *β*-glycerophosphate, 0.1 mM sodium orthovanadate, 0.1 mM phenylmethylsulphonyl fluoride, 5 *μ*g ml^−1^ leupeptin, 1 *μ*g ml^−1^ aprotinin, 0.2% Triton X-100, 0.3% IGEPAL). In all, 50 *μ*g of cell extract was separated by SDS–PAGE, transferred to nitrocellulose and membranes probed with any of the following: *α*-CHK2 Thr68-P (Cell Signalling, USA), *α*-CHK2 (N17, H300, Santa Cruz, USA), *α*-CDC25A F6 (Santa Cruz, USA), *α*-PP2A-A*β* (C-20,Cell Signalling, USA), *α*-p53 D01 (Novacastra Laboratories, UK), *α*-p53 Ser15-P (Cell Signalling, USA), polyclonal *α*-ATM (residues 250–522) (Chenevix-Trench *et al*, 2002).

## RESULTS

### Genotyping and LOH analysis

*CHEK2* 1100delC heterozygosity was identified in two out of 300 (0.6%) of index cases, in two families. Both of these families fitted Criteria 1B. All available male and female relatives (*n*=18) were then genotyped for the variant. Family 0011.00.005 had three cases of breast cancer, two of which were bilateral. Only these two bilateral cases were available for genotyping, and only one was a carrier of 1100delC. Family 0026.00.003 had three cases of breast cancer, two of whom (one with bilateral and the other with unilateral disease) were carriers. However, the variant was inherited from a parent with no personal or ancestral history of breast cancer, and not from the parent with an affected sister. Neither of these families contained cases of male breast cancer or colorectal cancer. Two tumours were available for LOH analysis, of which one showed loss of the wild-type allele and the other of the variant allele (data not shown).

### The 1100delC mRNA transcript is present in LCLs at a reduced level compared to the wild-type CHK2 transcript

**Figure 1:**
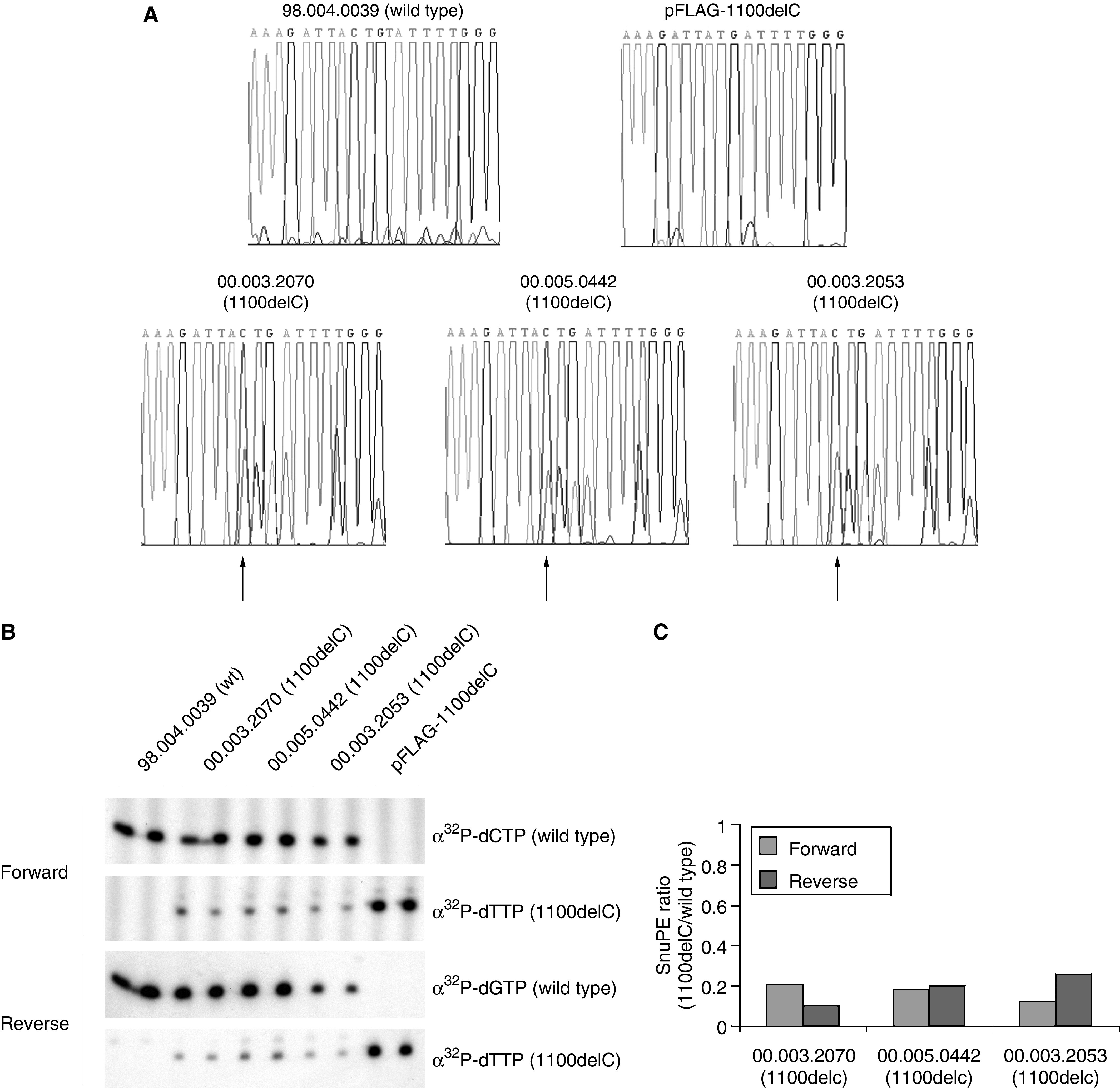
The 1100delC mRNA transcript is present in mutant LCLs at a reduced level compared to wild-type CHEK2. (**A**) cDNA sequence electrograms of the PCR products with arrows showing the position of 1100delC variation in heterozygous LCLs. Sequences are presented in the 5′–3′ direction. (**B**) Autoradiograph of SnuPE reactions from wild-type and heterozygous LCLs with both forward and reverse primers. (**C**) Quantitation of 1100delC expression in the three heterozygous LCLs. The mean of the duplicates was calculated and the level of the 1100delC mRNA was expressed as a ratio of the variant compared to wild type in both the forward and reverse directions.

To determine the functional effect of *CHEK2* 1100delC truncation, we established LCLs from three female *CHEK2* 1100delC carriers, 00.003.2053 (unaffected at age 54), 00.003.2070 (unaffected at age 79 and no family history of breast cancer except in her daughter and granddaughter) and 00.005.0442 (breast cancer at age 52), as well as LCLs from healthy wild-type individuals (98.004.0039) as controls. To test whether mRNA encoding *CHEK2* 1100delC was present in the LCLs, cDNA was prepared from wild-type and heterozygous LCLs, and a 190 bp region covering the open reading frame of *CHEK2* 1100delC was amplified. Direct sequencing of the product confirmed the existence of mutant transcript although its level appeared to be markedly reduced compared to the wild-type transcript (Figure 1A).

We then used SnuPE to compare the relative levels of the 1100delC mRNA to wild-type mRNA in the heterozygous LCLs. PCR products from the control (98.004.0039) and heterozygous LCLs extended the forward primer with ^32^P-dCTP and the reverse primer with ^32^P-dGTP, which is specific for the wild-type transcript (Figure 1B). Using ^32^P-dTTP, which is specific for the 1100delC transcript, the forward and reverse primers were only extended in the heterozygous LCLs (Figure 1B). When plasmid DNA containing the 1100delC mutant was used as template, the primer is extended only by ^32^P-dTTP and not ^32^P-dCTP or ^32^P-dGTP.

Densitometric analysis of the results obtained from the three heterozygous LCLs showed that the level of the 1100delC transcript was approximately 20% of the normal transcript (Figure 1C).

### CHK2 1100delC protein is not detectable in heterozygous LCLs

The 1100delC mutation induces a frameshift starting at codon 366 with subsequent truncation of the protein at amino acid 380, resulting in a protein product of approximately 52 kDa (*in silico* analysis using SwisProt). Antibodies directed against the N-terminal regions of CHK2 (N17 and H300) were used to analyse cell extracts from heterozygous LCLs for full-length CHK2 (62 kDa) and the truncated protein product (52 kDa). Only the full-length CHK2 protein was detectable by Western analysis and immunoprecipitation (data not shown).

**Figure 2:**
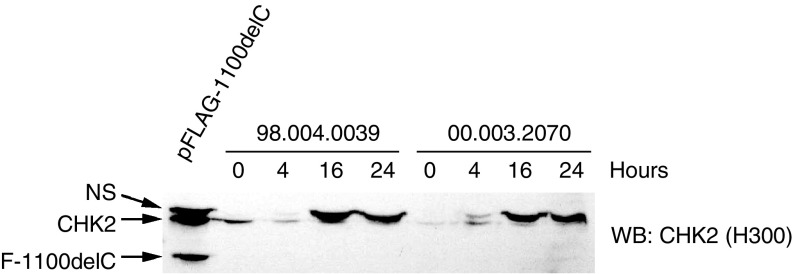
CHK2-1100delC is not detectable after proteasome inhibition. The indicated LCLs were untreated (0) or treated with 10 *μ*g ml^−1^ of ALLN for indicated times. Cell extracts were resolved by SDS–PAGE and immunoblotted with the CHK2 (H300) antibody. The stabilised band (top) is a nonspecific (NS) band detected with this antibody.

The absence of the truncated protein suggests that it is either unstable or not translated. To evaluate protein stability, one control (98.004.0039) and one heterozygous 1100delC LCL (00.003.2070) were treated with 10 *μ*g ml^−1^ of the proteasome inhibitor, ALLN, and harvested at 0, 4, 16 and 24 h after treatment. Cell lysates were prepared and analysed by Western blotting with the H300 CHK2 antibody. Cell extracts from 293T cells overexpressing FLAG-tagged CHK2-1100delC were used as a positive control to detect the truncated protein. During the course of treatment with ALLN, the levels of endogenous, wild-type CHK2 did not increase in either the control or heterozygous LCLs. Also, no truncated protein was detected in the CHK2-1100delC heterozygous LCL (Figure 2), suggesting that the truncated protein is not translated. However, it remains possible that a small proportion of CHK2-1100delC is present in these cells, but below the sensitivity of our detection method.

### Response of the 1100delC LCLs to DNA damage

**Figure 3:**
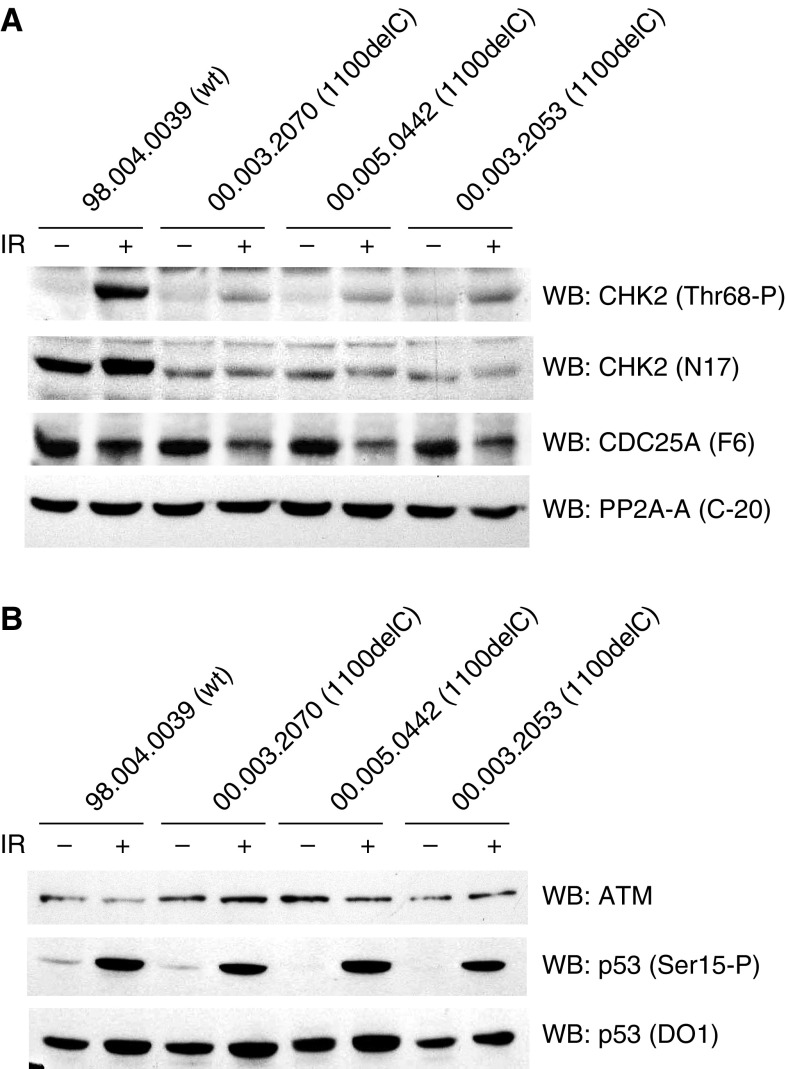
Response of the heterozygote 1100delC LCLs to DNA damage. (**A**, **B**) Lymphoblastoid cell lines from healthy control and heterozygous individuals were either mock-treated or treated with 6 Gy of IR and harvested after 30 min. Cell extracts were prepared and immunoblotted with the indicated antibodies.

To determine if the response of CHK2 to IR is impaired in heterozygous LCLs, phosphorylation of CHK2 on Thr68 and degradation of its downstream target CDC25A were studied by Western blot analysis of cell extracts prepared before, and after, exposure to 6 Gy of IR (Figure 3A). Three heterozygous 1100delC LCLs were compared with three control cell lines (data are shown for only one representative control). As expected, the control cell lines showed a marked phosphorylation of CHK2 after IR, whereas heterozygous LCLs from 1100delC carriers exhibited a markedly reduced phosphorylation of CHK2 Thr68. This reduction in the level of Thr68 phosphorylation after IR is presumably due to the reduction in the amount of total full-length CHK2 protein seen in these cells, detected with the CHK2 N17 antibody (Figure 3A), compared with PP2A, a loading control.

Owing to the reduced level of endogenous CHK2 in the heterozygous LCLs, we also investigated the downstream effects of CHK2 activation. CHK2 is involved in the intra-S-phase checkpoint that, in response to IR, results in degradation of CDC25A (reviewed in Bartek and Lukas, 2003). We evaluated CDC25A degradation after IR in wild-type and heterozygous LCLs, but found that there was no significant difference between them (Figure 3A), which indicates that signalling downstream of CHK2 is not compromised in heterozygous LCLs.

To ensure that the reduction in Thr68 phosphorylation of CHK2 was not due to an ATM defect upstream of CHK2, but was only a result of the decreased levels of CHK2, we investigated ATM phosphorylation of p53 at Ser15 as a measure of *in vivo* ATM activity (Banin *et al*, 1998; Canman *et al*, 1998; Khanna *et al*, 1998). Phosphorylation of p53 on Ser15 was similar between the control and heterozygous 1100delC LCLs, as was the level of ATM in these cells (Figure 3B). In addition, the stabilisation of p53 after IR was normal in the heterozygous LCLs compared to controls (Figure 3B), an event that is attributed to CHK2 phosphorylation of p53 on Ser20 (Chehab *et al*, 2000; Shieh *et al*, 2000).

### Overexpression of the 1100delC mutant does not affect endogenous Chk2 phosphorylation

**Figure 4:**
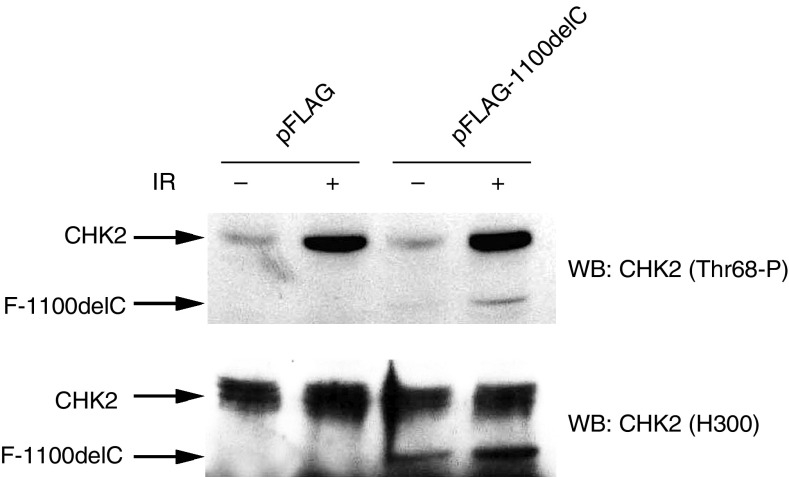
Overexpression of FLAG-CHK2-1100delC does not affect endogenous CHK2 function. 293T fibroblasts were transfected with either pFLAG or pFLAG-CHK2-1100delC and incubated for 36 h. The cells were either mock-treated or treated with 6 Gy of IR and harvested after 30 min. Protein extracts were prepared and Western blotting performed using the indicated CHK2 antibodies.

We performed functional analysis of the *CHEK2* 1100delC variant by ectopically expressing it in 293T cells to assess whether it undermined the phosphorylation and function of endogenous CHK2. Western blot analysis of cell extracts with anti-phospho Thr68 antibody revealed that the overexpression of the variant (detected with the H300 antibody) did not interfere with phosphorylation of endogenous CHK2 after IR, compared to vector-only transfected cells (Figure 4). The variant protein is clearly overexpressed, but is phosphorylated on Thr68 at markedly reduced levels compared to that of wild-type, endogenous CHK2.

Dimerisation is proposed to represent an important step in the regulation of CHK2 activity (Ahn *et al*, 2002; Xu *et al*, 2002). However, we were not able to detect dimerisation of variant protein with the wild-type CHK2 in cells that ectopically expressed both proteins (data not shown), indicating that the truncated protein is not likely to affect wild-type CHK2 activity in this way, even if it is expressed at very low levels (undetectable with our methodology).

## DISCUSSION

The *CHEK2* 1100delC variant has been reported in 30 out of 718 (5.1%) multiple-case families from Europe and the USA (Meijers-Heijboer *et al*, 2002). The frequency of the variant in controls ranges from about 1.3% in the Netherlands and Finland to 0.52% in UK and to about 0.2% in Germany (*CHEK2* Breast Cancer Case-Control Consortium, 2004). In Australia, the prevalence of the mutation is lower in 10 out of 1474 (0.68%) female breast cancer cases and one out of 736 (0.14%) controls (*CHEK2* Breast Cancer Case-Control Consortium, 2004). This study shows that only two out of 300 (0.6%) of index cases from multiple-case breast cancer families from Australia carry the variant.

*CHEK2* 1100delC has been reported in 18% of Hereditary Breast and Colorectal Cancer families from the Netherlands (Meijers-Heijboer *et al*, 2003). Our study of index cases from multiple-case breast cancer families included 152 who had first-, second- or third-degree relatives with colorectal cancer, as well as seven with male relatives affected with breast cancer, but the *CHEK2* variant was not identified in any of these index cases. Both families that carried the *CHEK2* 1100delC variant contained cases of bilateral breast cancer. However, bilateral cancer is not unusual in the multicase families recruited by kConFab, and the association between CHEK2 1100delC may be coincidental.

As only two families were identified with the *CHEK2* 1100delC variant, there was insufficient power to analyse the penetrance of the variant in these families, or to carry out any histopathological or clinical follow-up studies. Loss of heterozygosity analysis showed that in one tumour loss of the wild-type allele occurred, and in the second tumour there was loss of the variant. This is consistent with other reports that loss of either the wild-type or variant allele can occur (Vahteristo *et al*, 2002; Oldenburg *et al*, 2003).

Although a number of genetic studies have been carried out with *CHEK2* 1100delC allele, to date no functional analysis has been performed using heterozygous LCLs. In the present study, we examined the mRNA and protein expression of CHK2 1100delC variant. Mutant mRNA represented about 20% of the total CHK2 transcript, demonstrating that variant message is present but may undergo nonsense-mediated decay. Notably, we were unable to detect 1100delC protein in heterozygous LCLs although several methods (Western blotting, immunoprecipitation and proteasome inhibitor treatment) were employed. The amount of total CHK2 protein, and phosphorylation of CHK2 on Thr68, in heterozygous LCLs was reduced to about half compared to wild-type LCLs, suggesting that 1100delC variant may act simply by haploinsufficiency. The increased breast cancer risk associated with *CHEK2* 1100delC may therefore be attributed to a threshold level of phosphorylation of CHK2 below which cells become more susceptible to other genetic and environmental factors promoting tumorigenesis. Consistent with this, we were not able to demonstrate any dominant interfering affect of 1100delC variant when ectopically expressed in 293T cells. Furthermore, although full-length CHK2 expression and Thr68 phosphorylation were considerably reduced in heterozygous LCLs, degradation of Cdc25A (a downstream target of CHK2) was normal. This may be due to compensation of CHK2 function by CHK1, which has redundant functions in targeting the same substrates. Compensation of the 1100delC defect in CHK2 by CHK1, or any other mechanism, might explain the rather low breast cancer risk associated with the *CHEK2* variant, compared to that associated with truncating mutations in *BRCA1* or *BRCA2.*
